# Ethical challenges and lack of ethical language in nurse leadership

**DOI:** 10.1177/09697330211022415

**Published:** 2022-05-27

**Authors:** Anne Storaker, Anne Kari Tolo Heggestad, Berit Sæteren

**Affiliations:** Oslo Metropolitan University, Norway; VID Specialized University, Norway; University of Oslo, Norway; Oslo Metropolitan University, Norway

**Keywords:** Ethical challenges, ethical language, moral distress, nurse leadership

## Abstract

**Background:**

In accordance with ethical guidelines for nurses, leaders for nurse services in general are responsible for facilitating professional development and ethical reflection and to use ethical guidelines as a management tool. Research describes a gap between employees’ and nurse leaders’ perceptions of priorities.

**Objective:**

The purpose of this article is to gain deeper insight into how nurses as leaders in somatic hospitals describe ethical challenges.

**Design and method:**

We conducted individual, quality interview with 10 nurse leaders, nine females and one male nurse aged 34–64 years. We used a hermeneutical approach to analyse the data.

**Ethical considerations:**

The participants received oral and written information about the study. Participation in the study was voluntary, and the participants were given the opportunity to withdraw. All of them gave written consent. The Norwegian Centre for Research Data approved the research project. In addition, the head of the hospitals gave permission to conduct our study.

**Findings:**

Four main areas were identified: deficient ethical language, conflicting demands on nurse leaders regarding staff management, concerns regarding young nurses’ ethical consciousness and restricting factors on the creation of a climate of ethics. The nurse leaders experienced considerable pressure. An unexpected finding was the lack of – and even disregard for – an ethical language.

**Discussion and conclusion:**

It is crucial to recognise ethics in all types of nursing approaches and to make it explicit. Ethical language must be implemented in nursing education. It must be recognised and used in clinical practice.

**Recommendations:**

We recommend further research be conducted into how nurses understand the concept of ethics and how to incorporate ethical principles into clinical nursing and nurse leadership.

## Introduction

In accordance with ethical guidelines for nurses, leaders of nursing services have a particular responsibility to facilitate professional development and ethical reflection and to use professional ethical guidelines as a management tool.^1^ This reflects the important role that nurse leaders play in enabling their staff to provide high-quality care within the rules and framework of their organisation, in addition to creating an organisational climate and conditions for ethical practice. This means that they have responsibility for facilitating an ethical climate among nurses on the wards. Ethical climate may be understood as a common perception of how to deal with ethical challenges and of what behaviour is considered by patients and their organisations to be ethically appropriate.^2^ As far as ethical leadership is concerned, nurse leaders are ethical role models for their employees,^3^ and there is an assumption that they also have an obligation to provide ethics support to their employees. When it comes to patient care, they are responsible for the quality of patient care delivered on their wards. This means that nurse leaders have responsibility for managing ethical leadership at micro, meso and macro levels,^3^ and may experience competing needs and values which may create ethical challenges on several levels. In this study, an ethical challenge is defined as ‘any situation that requires ethical deliberation or ethical decision making, or a conflict of moral values’.^4^ The extent to which nurse leaders are aware of, understand and solve challenges like these may affect the ethical climate, the caring culture and, hence, the quality of care delivered on the wards.^5^ According to Storch et al.,^6^ ethical leadership involves responsibility to engage in ethical behaviour and build a moral community. It is important to gain knowledge about ethical challenges that may arise in nursing leadership and about how nurse leaders understand their role and responsibilities in relation to these challenges.

### Literature review

According to Denier et al.,^7^ nurse leaders play an important role in creating a context for ethics to take place. They are important role models, and how they express their own values is significant in ethical leadership. There are, however, several obstacles which may challenge nurse leaders in managing ethical leadership, such as organisational culture and a lack of power and authority as nurse leaders.^7^ Feelings of powerlessness among nurse leaders is also highlighted in an article by Sieloff.^8^

Storch et al.^6^ found that registered nurses often encountered a lack of ethical leadership and that nursing leaders lacked sufficient support and guidelines to secure ethical leadership. They also found that front-line nurses lacked support from their nurse leaders in order to provide safe, compassionate and ethical care. This lack of support led to moral stress for the nurses. Research also shows that ethical, cultural and marginal problems may challenge ethical leadership and, hence, lead to ethical stress among leaders and nurses.^9^ Other studies show that good ethical leadership may have an effect on the ethical climate, which in turn may contribute to affective commitment to the organisation and less turnover.^2^ In a previous study we conducted, which focused on ethical challenges experienced by nurses, we found that there was little room for ethical reflection and that nurses experienced a lack of support from their nurse leaders when dealing with ethical challenges. Another study^10^ made the same findings and argued that lack of an ethical climate may contribute to moral distress among nurses. This indicates the responsibility nurse leaders have for preventing moral distress among nursing staff by promoting a good ethical climate on the wards.^2^ Devik et al.^11^ suggest that nurse leaders in long-term care found ethical leadership important but challenging. Time constraints and organisational obstacles were cited as reasons for why the work was found challenging.^11^

Previous research suggests that conflicts between budgetary constraints on one hand and maintenance of quality in health and care services on the other are perceived as demanding for nurse leaders.^12^ Skirbekk et al.^12^ argue that there is a gap between employees’ and leaders’ perceptions of which priorities are most important. The staff faced challenges in prioritising their time. Consequently, they were unable to attend to patients as much as they would like. However, top leaders were more concerned with keeping within budgets and maintaining appropriate patient flows.^13^ Lindy and Schaefer^14^ found that most nurse leaders can encounter poor behaviour among their employees and that many find it ethically challenging to address such situations in a way that safeguards employees. A study by Foss et al.^15^ showed that nurse leaders may experience conflicts between care values and external frameworks for which they are responsible. Such external frameworks can be financial or structural in nature.^16^ Norwegian somatic hospital settings have different levels of leadership. The nurse leaders in this study were ward leaders.

### Purpose

The purpose of this article is to gain deeper insight into how nurses as leaders in somatic hospitals describe ethical challenges.

## Methodology

### Research design

This study has a hermeneutical approach inspired by Gadamer’s ontological view of hermeneutics.^17^ Understanding is fundamental in hermeneutics, and since the purpose of the study is to gain an understanding of the ethical challenges faced by nursing leaders in hospitals in their daily work, we found it appropriate to choose a hermeneutical approach. The goal in hermeneutics is to reach as deep an understanding as possible. Interpretations that lead to understanding are not necessarily correct but may be reasonable. Gadamer did not develop a method of interpretation, but he presented some key concepts in the hermeneutical quest for knowledge in the hermeneutical circle: openness to the text, questioning and answering, considering the parts of the text and the whole, and the fusion of horizons. In the fusion of horizons, the researcher’s preunderstanding and the text merge, and a new understanding emerges. The intention in hermeneutics is to search for the truth that lies behind the immediate reality.

### Study setting, sample and method of data collection

We conducted qualitative interviews to collect data and to gain insight into ward nurse leaders’ experiences of ethical challenges. Since this is a Norwegian study, all the interviews were performed in Norwegian. Nine female nurses aged 34–63 years and one male nurse aged 44 years participated in the study. Nine were nurse leaders on clinical wards, while one was head of an outpatient facility. The interviews were carried out individually and transcribed verbatim. The data were stored in accordance with the regulations of the Norwegian Research Council and Oslo Metropolitan University, on an encrypted memory stick kept in a lockable cabinet. Data were collected during spring and early summer 2019. Most of the patients on the wards had been diagnosed with different types of cancer. The personnel groups on the various wards varied in number. Some wards had a very large number of employees due to mergers in recent years. When it comes to education beyond basic and leader education, three of the nurse leaders held master’s degrees.

Fourteen nursing leaders were invited and 10 agreed to participate. An interview guide based on the purpose of the study was prepared, but an intention during the interviews was to get the leaders to speak openly and freely about their experiences. The interviews took place at the leaders’ offices and lasted from 1 to 1.5 h. The interviews were taped and transcribed and form the text for the analysis and interpretations. The first author attended all the interviews while the other authors each attended half of them.

### Data analysis and interpretation

The analysis was performed in accordance with Kvale and Brinkmann’s^18^ three levels of analysis: self-understanding, common sense and theoretical understanding. Data from the interviews were read several times to gain an overview of the data material. To structure the data further, we used a matrix where the first heading was: What do the participants say? This question summarised the level of self-understanding that was illustrated by quoting from the text. The next question in the matrix was, What is the meaning of what the participants say? This represented a common understanding of the issues that went beyond the meaning of the participants. Finally, the text was read carefully to allow for identifying the hidden meaning beneath the words. In this third step – the theoretical level – one makes interpretations beyond the common-sense level. This third level is arrived at after repeatedly reading, interrogating the text, answering the questions, and moving back and forth between the parts and whole. Every step was assessed according to the purpose of the study to ensure that we retained the focus. Preunderstanding is essential in hermeneutics, both preunderstandings to gain new understanding and preunderstanding as a potential obstacle to understanding that is created by one’s own bias. Since all the researchers are familiar with the research context, awareness of and challenges to their own preunderstandings were important considerations during the research process. Throughout the analysis and the interpretation process, the research group discussed themes and reached consensus. The interpretation process resulted in four main themes.

### Ethical considerations

The participants were recruited by the authors. Once they agreed to participate, the participants received written information about the study and were contacted to arrange a time and place for the interviews. The participants gave their written consent before the interviews. They were informed in writing of the principle of voluntary participation, the duty of confidentiality and anonymity, and the opportunity to withdraw from participation without giving any reason. The study was approved by the Norwegian Centre for Research Data, project number 234670.

## Results

Our analysis produced four main findings regarding the ethical challenges nurse as leaders deal with in somatic hospitals: (1) Deficient ethical language; (2) Conflicting demands on nurse leaders regarding staff management; (3) Concerns regarding young nurses’ ethical consciousness; and (4) Restricting factors in creating a climate of ethics.

### Deficient ethical language

An unexpected finding was that ethics as a concept seemed to be unfamiliar and rarely used on the wards. Most of the participants did not use ethical terms or ethical language. Thus, it may be difficult to interpret exactly how they understood it. However, two participants who held master’s degrees in values-based management described the concept as complicated and wanted to promote ethical thinking among their staff. They approached this by selecting what values they wanted their staff to adopt.

Some participants described ethics as an alienating term which they disliked using, while others found it vague and difficult to understand. All of them talked about situations where ethical issues were obvious, but they did not address them as ethical situations. Nor did they use theoretical concepts such as paternalism and dignity while describing patients’ situations. It seemed as if they were somewhat reluctant to use ethical terms. Thus, it can be interpreted as if they understood ethics to be integrated into professional nursing without regarding it as a separate aspect to reflect on. One participant characterised the term ‘openness’ as important to her. However, she emphasised ‘openness’ as a word, not as a value. Another participant said she sometimes felt that ethics was everywhere and should be used to resolve everything. She said she became uncomfortable from that kind of thinking. A third participant said she had to prepare herself by reading ethical theory before the interview in order to understand potential questions. She found the concept strange and difficult to understand. She said that she never dealt with it consciously in her daily practice. This may indicate that ethics was not emphasised as much as, for example, medical issues and thus was not specifically highlighted.

Nevertheless, the participants wanted the staff to have a high level of competence and to meet patients with the following attitude: ‘What is important to you?’ One nurse leader reported the following incident:

A patient asked to speak to me as leader. She had asked a nurse to help her in the shower because she felt tired and depressed. The nurse had replied that she should be able to manage herself and left the room. The patient found this very hurtful. I understood the patient’s reaction and decided to talk to the nurse personally in my office. I told her the patient’s story and saw that she realised immediately that she had made a mistake. She went back to the patient and apologised for her behaviour. (4)

The example above confirms that this leader was concerned about the patient’s vulnerability. In addition, she emphasised that the employee should meet the patient in a caring manner. The fact that she called the employee in for a conversation shows that she addressed issues to add overall values such as honesty and justice, which in turn shows what we would call ethical leadership.

As a group, the participants seemed to use a mixture of vernacular and medical language. They seem to talk about challenges in general without specifying ethical, practical or medical challenges. One nurse leader explained,

Ethics is, after all, a big word that you can think a lot about, about doing right or wrong, and about doing things in the right way, to do what is morally right for you in accordance with the values you believe in. It’s important for me to be a good role model. But I don’t use the word ‘ethics’ that much. I prefer to use the term ‘value’ and to communicate values that are important to me and what values I want my nurses to have. (7)

To summarise, we found that the participants were engaged in ethical issues without using an ethical language. Based on the statement that there is ethics in everything, they seem to consider ethics as part of general nursing though without a specific and necessary language. In addition, the fact that ethical situations were not referred to as such may indicate that ethics as a professional issue was not given much importance on some of the wards.

### Conflicting demands on nurse leaders regarding staff management

All the participants replied spontaneously that they enjoyed being nurse leaders. They seemed to be inspired by the ideal of doing something useful for other people. They underlined the importance of optimal patient care as an aim in their management practice. They expressed a desire to influence and create a meaningful culture for patients, next of kin and staff alike. Furthermore, they appeared to want an open culture where professional and moral issues could be discussed in order to achieve their goals. They expressed an empathic approach. Moreover, they were concerned about communication in general and underlined the importance of talking in a caring way to patients and of talking respectfully and openly to other members of staff. One nurse leader expressed his experience as follows:

I think it is very exciting with management because you may be able to do something to influence how the staff experience their work in a positive way. I hope and think that will be an approach that can influence caring for the patient in a positive way. (10)

Another nurse leader described her awareness of how leadership provides power due to the organisational structure:

I feel I have a lot of power as a nurse leader. It scares me in a way, because if I should use my power wrongly. I think it’s important to use reflection as a tool to make the right decisions. (2)

All the participants reported that personnel matters represented their most challenging task as nurse leaders. They had to conduct employee interviews with everyone once a year, and they demanded of themselves that each employee should be treated fairly. Justice and equality appeared to be values to which the participants gave high priority. They spent time and energy on being fair. However, the principle of fairness seemed to be associated with working conditions such as shift rotas. They wanted everyone to feel that their requests for time off were granted and that well-paid shifts were evenly allocated. All participants reported some sick leave. They openly admitted that some staff members were easier to work with than others, though they would not allow personal preferences to influence their decisions. One nurse leader said,

They all have their different needs, needs to be seen in their own way, and it’s not always possible to work that out. And you must be fair. It’s important to me that they feel that I treat them fairly, whether it be about shift rotas or taking a day off. And about how I allocate resources to the different groups, because the ward is divided into three groups here. That’s perhaps the most difficult one. Hmm. But also, very interesting (1).

Many employees complained of heavy workloads and staff shortages. One nurse leader expressed this as follows:

I just have to tell them that, yes, I understand that you don’t have time for it, you just have to remember to prioritise. And that is caring, administering medication, and then let’s just take that teaching another time and that tomorrow’s a new day. (7)

However, most of the patients were seriously ill and needed continuous care, which created a highly stressful situation for the staff. Because of this, they organised supervised groups with external supervisors. They did not participate themselves. The mandate for the groups was to provide general nursing professional guidance, not particular guidance in ethical issues. Nevertheless, many, especially the young and newly trained nurses, needed individual follow-up from the nurse leaders.

To summarise, one goal for the leaders was to provide optimal care and treatment to patients. Another goal was to have a professional, competent and well-functioning staff. The employees should feel fairly treated and should be satisfied and committed. Owing to time shortages and high stress levels, these goals came into conflict and resulted in some of them not always being reached, such as having satisfactorily competent staff on duty.

### Concerns regarding young nurses’ ethical consciousness

The nurse leaders expressed concerns about changes they were seeing among the young nurses compared to previous years, and that they seemed immature and less reflective. Several of the participants emphasised that they were distressed about young nurses’ ability to reflect on ethical issues such as understanding vulnerable patients. Nevertheless, the young nurses appeared empathetic, and eager to enhance their professional and personal development.

One nurse leader expressed her view as follows:

I think maybe some people take a long time becoming mature enough to realise that there is a world outside themselves. Yes. Yes. I don’t think they’re at all aware of the ethical issues around them. (1)

Furthermore, she attributed their attitudes not only to their age, but also to generational changes and to changes in society in general. They seemed to consider their job as an opportunity to make new friends and have fun. Moreover, they no longer seemed to subscribe to the idea of nursing as a calling.

A common feature among the young nurses appeared to be a practical approach. They wanted to be efficient, go to the patient to get the work done, and then return to the staffroom and, often, to the computer. Some nurse leaders found that the younger nurses tended to be more concerned about informing patients than about asking them questions about their worries and that they often complained of being tired after only a few hours at work. One nurse leader reported that she used to recommend young and newly trained nurses to spend more time with their patients, do something for them or just talk to them, because that would lead to more meaningful use of their time and would probably prevent them from feeling tired. Another nurse leader expressed her view as follows:

The young nurse asked: ‘Why should I go into his room and talk to him?’ I replied: ‘Why not? When I go into the kitchen to get my morning coffee, I can hello to the patient, how are you and things like that. You can do the same’. (6)

However, the young nurses spent time learning work routines such as medical procedures, drug handling and documentation. They seemed to be less focused on holistic nursing and the hygienic aspects of keeping rooms clean and tidy. Some of them seemed to forget the importance of changing linen and supplying fresh towels. They also forgot to empty the garbage cans and clean the bedside tables unless they were asked to do so. The nurse leaders worried how these attitudes might affect patients’ well-being. One nurse leader said,

I think they got an ‘aha experience’ because we had taken some pictures of an ordinary patient room. A person, who was covered over, was lying in bed. On the bedside table there were dirty cups and other waste items. The garbage can on the floor was overfilled. The linen was not clean, and the bed was untidy. This situation resulted in constructive discussions and really improved attitudes. (7)

Another participant (1) emphasised that she did not perceive the young nurses as a homogeneous group, but rather as young people with individual skills. She explained that she decided to enjoin a young nurse who she thought might benefit from a challenge to be the primary contact for a 14-year-old boy who was terminally ill with cancer. The young nurse accepted the challenge. The participant summed up the situation by stating that the young nurse had treated the patient in a competent way and had realised what a difference she had made for him. Her professional attitude changed after this experience, and she seemed able to see the whole patient in a different and deeper way than before.

In sum, these findings confirm the nurse leaders’ ability and willingness to guide young nurses in giving high-quality patient care. Furthermore, the participants wanted to guide their younger staff in discovering both improvements and progress in their professional development. Again, this suggests that the nurse leaders had ethical insight and competence, despite their lack of ethical language and opposition to ethical thinking.

### Restricting factors in creating a climate of ethics

The participants described their work and duties as a constant squeeze in different directions. The experience of being pulled in different directions at the same time, such as budget meetings, challenging patient situations and vacant shifts, brought them little peace of mind. The frequent reorganisation processes implemented by Norwegian hospitals to make operations as rational and economical as possible could be understood as restricting factors in creating an ethical climate. Departments are merged into larger units with large staff groups. In addition, much of the building stock is old and run-down. Another problem was the lack of space and beds. Situations such as shortages of single rooms and having to let sick patients lie in beds in corridors appear to create difficult dilemmas such as breaches of confidentiality. One nurse leader said,

You’re always squeezed between the patients and having managerial resources. You have to report to your boss. And because you want to do the best for the patients and the staff…but then you have limitations in terms of budget. And then you have staff responsibilities and turnover. Yes, there are many things that place some kind of restrictions on where…how far you can go. (10)

As far as complex patient needs and medical treatment are concerned, there was an increasing demand for high levels of nursing competence. Procedures that used to be performed by physicians, such as cytostatic infusions, were now being performed by nurses. An additional pressure on nurse leaders was the constant introduction by management of new scoring and screening tools such as NEWS and ISBAR in order to improve the quality of patient care delivery. However, they found that the staff were engaged in using these tools. Requirements for documenting and reporting treatments were other time-consuming factors.

Economic regulations can affect daily operations in many ways with regard to patient travel between local hospitals and home. Even on discharge, patients are often very much affected by their illness, yet they are expected to travel by bus with facilitations such as a medically trained person who can provide help. A nurse leader expressed her concerns as follows:

Yes, it can be difficult. Especially when you are pressed for, well, funding and space and resources, but at the same time, my priority is patient treatment. So, if I must exceed my budget for someone to get what they’re entitled to or should get, then, then, for me, then, it’s basically a simple choice. (5)

When it comes to the everyday running of the wards, collaboration between nurse leaders and physicians in general seems constructive. However, it seems as if physicians and nurses do not communicate well enough in gaining a common understanding of concepts such as palliative care and overtreatment. They may not spend enough time agreeing on what is best for the patient in complicated situations like these. A severe ethical challenge appeared to be related to termination of patient treatment. Some of the nurse leaders perceived that the physicians were preoccupied with overtreatment, prolonging lives with cytostatic treatments and demanding surgical interventions. They also seemed less concerned with discussing such ethical issues with the nurses. One nurse leader said,

The physicians are concerned about palliative care, and they want the patients to have the best treatment. The challenge is that we disagree about what palliative care and pain treatment entail. (8)

To summarise, the sense of being squeezed was obviously demanding on the nurse leaders. In addition, senior management seemed more concerned with keeping within budgets than with creating an ethical climate.

### Summary of findings

The participants did not promote an ethical language. In fact, several of them seemed to disregard the need for it. Despite this, they emphasised ethical guidance, ethical leadership and the need to create an ethical climate, but without using ethical terms. This might be interpreted as a paradox. Furthermore, all of them felt that budgetary constraints and other duties imposed on them seemed to create an adverse pressure. All in all, these factors may have diverted the need for and recognition of ethical language away from conscious holistic thinking.

## Discussion

In the discussion, we would like to address ethical and professional challenges that may arise when ethical language is partly absent or not consciously present, and obstacles to ethical leadership. The position of nurse leaders and of nurse leadership may be illustrated as an octopus, that is, a core with several arms. The core symbolises the leader’s nursing ideals and leadership qualities, while the arms symbolise all the nurse leader’s responsibilities. In this context, it may be understood that having two arms is not enough to be a nurse leader.

### Ordinary problems versus ethical problem: the effects of a deficient ethical language

The nurse leaders as a group perceived the concept of ethics as vague and unclear. Neither they nor their staff seemed to recognise the concept. Despite their opposition to ethical language, they talked about ethical issues. We understood this to mean that they regarded ethics to be integrated into general nursing and not emphasised with a separate language. However, our findings confirm the nurse leaders’ ability and willingness to guide young nurses in giving high-quality patient care. Furthermore, the participants want to guide their younger staff members in developing professionally. Again, this suggests that the nurse leaders had ethical insight and competence which they did not appear to recognise themselves.

This can be understood as a paradox on one hand and as an underestimation of the importance of professional language and a disregard for associated theory on the other. The fact that ethical situations were not referred to as ethical may indicate that ethics as a subject area was often not considered important to discuss on the clinical wards. However, ethics in itself may be challenging because ethical issues can be difficult to express orally, especially if the ethical language is unfamiliar. In addition, the issues may sometimes be associated with moral correction, which many would likely avoid.

According to Nortvedt,^19^ as far as nursing is concerned, ethics is often a matter of having the right attitude and moral sensitivity in the particular situation and of focusing attention on what is at stake. Thus, it is natural to believe that patient care needs a rich ethical language to adequately describe incidents of vulnerability and moral nuances. In an interview,^20^ Kari Martinsen argues that professional language in nursing is characterised by financial terminology. Ethics is lost because nurses do what the working culture commands. The Norwegian financial system is based on diagnosis-related groups, a patient classification system that provides simplified descriptions of hospital activities and patient compositions that form the basis for funding.^21^

In one of her books, Kari Martinsen^22^ draws attention to moral judgement as a professional concept. She argues that language creates distance from the situation in which we are to act. This distance is important and gives us time to think before we act. She concludes that language conveys a common understanding of both our thinking and our actions. Action and thinking are intricately interwoven.^20^ We understand this from the way in which professional and nuanced language reflects the theoretical background on which our actions are based; it may provide courage and the opportunity for professional observation and argumentation.

Besides, one of our findings suggests that nurses and physicians do not collaborate well enough when it comes to ethical issues such as clarifying palliative care and nursing. There is reason to believe that a nuanced language would improve the nurse and the nurse leader’s ability to argue in challenging ethical issues and in general. A well-developed professional and ethical language may help clarify what nurses professionally think and contribute to more advanced argumentation. It may also contribute to increased professional confidence among nurses. Moreover, it will put ethical issues from the nurses’ as well as the physicians’ perspectives on the agenda when treatment is to be changed or terminated.

Makaroff^23^ argues that although literature on the unsayable has been developed primarily outside the discipline of nursing, exploration of the concept within nursing may help nurses to consider situations and experiences that are challenging, elusive and perhaps impossible for patients to articulate during their illness. It is a challenge to find words, terms and concepts that correspond with what is described. However, ethical theory is supposed to contribute to shedding light on difficult moral issues.^24^ Knowledge of ethics and ethical reflection may help individuals to become aware of their own attitudes and values. The nurse leaders with values-based master’s degrees seemed more familiar with the concept and with ethical thinking. However, they found that their employees showed no interest in gaining deeper insight into ethical knowledge. Nonetheless, they emphasised a wish to be role models for their employees, which is in accordance with Gallagher and Tschudin’s^3^ definition of an ethical leader as ‘one who demonstrates commitment to ethical practices in their everyday work and act as ethical role model for others’. This means becoming more aware of one’s own professional demeanour and appearance. According to Brown, ethical leadership is the demonstration of normatively appropriate conduct through two-way communication, reinforcement and decision making.^25^ Moreover, their findings support the idea that role modelling is related to ethical leadership. Having an ethical role model during one’s career is positively associated with ethical leadership. However, they suggest that the importance of career ethical role models is stronger for older leaders.^25^ Moreover, Makaroff et al.^26^ argues that ethical leaders are characterised as individuals with higher levels of ethical standards, integrity and verity. The nurse leaders in this study set high standards for care, though some of them seemed to disregard the importance of an ethical and professional language to express their standards.

There seems to be a need to raise awareness of the ethical language of nursing in general and in nursing leadership in particular. Ethical language must be implemented in nursing education and be recognised and used in practice. The aim is that nurse leaders themselves should realise and understand the need for a nuanced ethical language for describing their professional thinking and doing. In addition, acknowledge of this problem may contribute to improved professional approaches such as ethical understanding of and skills in ethical argumentation.

### The challenges of ethics

All the participants expressed that they found it interesting and challenging to be a nurse leader, despite feeling a constant squeeze. Their ethical challenges seemed to be related to the opportunity and ability to provide optimal patient treatment and care satisfactorily and with sufficient competence, and smooth cooperation with the top management. Hence, constant pressure from the hospital management to stay within budgets and prioritise effective reporting represented an additional source of stress. Skirbekk et al.^13^ report similar findings, where clinicians, both nurses and physicians, felt that their professional and patient-centred ideals were threatened because nurse leaders had become administrators, focusing on efficiency in terms of patient flows. Top management’s budgetary pressures seem to divert attention away from positive professional processes because communication between units is not good enough to construct common goals. A study of municipal healthcare^12^ reported similar findings, describing a battle between budgetary and caring perspectives. The caregivers were required to find a balance in this battle which naturally will increase stress.

Another aspect of the squeeze related to disagreement over how to deal with complex patient issues such as overtreatment and termination of treatment. The nurse leaders described these issues as ethical challenges for patients and staff alike. The problem created stress for the nurses because they found that many patients suffered unduly from the treatment without recovering. Two nurse leaders blamed the physicians for ignoring the nursing assessment in these cases. They emphasised that improved collaboration with and communication between physicians and nurses would reduce the problem. According to Kramer and Schmalenberg,^27^ forging collegial nurse–physician relationships is a process that needs standards, just as we have outcomes and standards. Consideration should be given to implementing measures that describe the nature, steps and activities for improving care quality through enhanced partnerships. A good and safe relationship between physicians and nurses is necessary in order to see patients and individuals and to act in the patient’s best interests. A nuanced language will probably improve the ability to argue clearly. However, we do not know for sure whether physicians have an ethical language. In contrast to medicine, ethics is not an exact science. By expressing oneself ethically, one may expose one’s own insecurities, which may feel unfamiliar and challenging for nurses, nurse leaders and physicians on clinical wards. A common ethical language might lead to both improved communication and collaboration. Moreover, it is important that nursing education must emphasise factual knowledge, such as medical knowledge, and training in ethical awareness, ethical understanding and expressing something in an ethical manner.

When it comes to consideration of employees, the participants emphasised fairness, openness and safety as important values. Most of them had large staffs, varying from 60 to 80 employees. All of them described this part of their work as the most morally demanding. They perceived having control of a large team of employees as a heavy and time-consuming responsibility. They seemed to want to make each nurse feel that he or she was treated fairly and with respect. This finding is interesting, considering the finding in other studies^6,10^ that nurses perceive their nurse leaders as invisible, distant, not present or supportive. The study by Storaker et al.^10^ showed that nurses perceived leaders to be less involved in daily problems such as prioritising tasks and collaborating with physicians. Storch et al.^6^ showed similar findings, saying that front-line nurses lacked support from their nurse leaders in providing safe, compassionate and ethical care. This lack of support led to moral stress for the nurses. Nonetheless, the participants in our study appeared to believe that their approach might lead to a pleasant and secure environment and focused on the overall goal: the patient’s best interests. On the contrary, their approach could have significance for their own role in making the relationship between leader and employee safe. Hence, the responsibility involves professional as well as ethical development.

Another emphasised value was appreciation of young, newly trained nurses. Some of the nurse leaders described their relationships with these nurses as challenging. They perceived them to be both idealistic and dedicated to their job, yet they seemed to struggle with engaging with their patients. They appeared to be concerned about the procedures and had difficulty in seeing the whole patient. This can be understood to mean that they were unable to see whether a patient is vulnerable. Moreover, according to Benner’s middle level of developmental moral maturity, the patient appears as a total human being for the nurse, who is beginning to identify needs beyond those of the illness and the treatment.^28^ The newly trained nurse has the knowledge and the know-how but not enough in-depth experience to deal with the complexities of nursing. She or he will need guidance and support.^28^ In fact, this might indicate that too much is expected of young nurses when it comes to both understanding and communicating more deeply with patients. They will need ethical guidance to develop further. However, we found that the nurse leaders themselves guided the young nurses in both practical and ethical matters without regarding these matters as ethical. On the contrary, it may be challenging to give professional guidance in ethical issues when you yourself do not have a conscious ethical language. In order to improve nursing language, it is crucial that nurses encounter an ethical language that they understand and can use in their education and in their clinical practice.

To summarise, the nurse leaders addressed many ethical challenges, such as difficult patient issues, demanding collaboration with physicians and budget issues. In addition, relatively high staff turnover meant they were constantly having to hire and train young, newly trained nurses. They felt they were being pulled in several directions and did not have enough time to pay attention to creating an ethical climate.

## Conclusion and recommendation

Health professionals will assume that nurse leaders have developed an ethical language and are highly trained in ethical thinking. Our study shows that some nurse leaders clearly lack and underestimate these skills. Professional and ethical issues are closely interlinked. We argue that it is crucial to recognise ethics in all nursing approach and to make it more explicit. Nursing actions are complex and extensive, and to better understand them, it is important to distinguish between the practical and the ethical problems. We believe that a nuanced ethical language is important for expressing professional nursing in a thorough manner. This can make nurses feel safer and better able to discuss ethical challenges. Thus, there is a need for hospitals and universities to provide ethics education for nurse leaders. We recommend further research into how nurses understand the concept of ethics and how to implement it in clinical nursing and nursing leadership.

## Methodological considerations

Although all the participants in this study were ward nurse leaders, they varied in age, ethical competence, years of experience as leaders and number of staff. This provided a rich data material and a variety of descriptions of ethical challenges. The participants in this study represented only one level of leadership in a hospital, that is, ward leaders. This is a limitation, since nursing leadership in hospitals represents hierarchical levels of leadership. Interviewing nurse leaders on another level would probably have resulted in different answers. However, this was a conscious choice because we wanted nurse leaders who were directly responsible for patients and whose employees were providing first-line patient care. The fact that all the leaders in the study held similar positions may have strengthened the validity of the study.

The nurse leaders in this study were responsible for wards caring for patients with different types of cancer. This is a limitation. More variation in the patient population might have strengthened the study and provided a broader and more nuanced picture of the ethical challenges nurse leaders deal with in their daily practice.

Only one of the participants was male. A higher proportion of male participants in the study might have produced different results. Even though the participants were encouraged to speak freely about their experiences of ethical leadership, an interview guide was applied to focus on certain questions and themes and thereby ensure the reliability of the data which were collected. The first author attended all the interviews together with one of the other authors. This ensured continuity in the process and strengthened reliability. The validity of the study was strengthened by the fact that all three researchers analysed the data material and discussed the interpretations until they reached consensus.

Although the sample was small (10), the main category emerged after only a few interviews. However, a larger sample and more interviews with each leader would have been a strength in the study and might have produced more nuances in the findings.
